# Broadband Criticality of Human Brain Network Synchronization

**DOI:** 10.1371/journal.pcbi.1000314

**Published:** 2009-03-20

**Authors:** Manfred G. Kitzbichler, Marie L. Smith, Søren R. Christensen, Ed Bullmore

**Affiliations:** 1 Behavioural & Clinical Neurosciences Institute, Departments of Experimental Psychology and Psychiatry, University of Cambridge, Cambridge, United Kingdom; 2 MRC Cognition and Brain Sciences Unit, Cambridge, United Kingdom; 3 Clinical Unit Cambridge, GlaxoSmithKline, Addenbrooke's Hospital, Cambridge, United Kingdom; John Radcliffe Hospital, United Kingdom

## Abstract

Self-organized criticality is an attractive model for human brain dynamics, but there has been little direct evidence for its existence in large-scale systems measured by neuroimaging. In general, critical systems are associated with fractal or power law scaling, long-range correlations in space and time, and rapid reconfiguration in response to external inputs. Here, we consider two measures of phase synchronization: the phase-lock interval, or duration of coupling between a pair of (neurophysiological) processes, and the lability of global synchronization of a (brain functional) network. Using computational simulations of two mechanistically distinct systems displaying complex dynamics, the Ising model and the Kuramoto model, we show that both synchronization metrics have power law probability distributions specifically when these systems are in a critical state. We then demonstrate power law scaling of both pairwise and global synchronization metrics in functional MRI and magnetoencephalographic data recorded from normal volunteers under resting conditions. These results strongly suggest that human brain functional systems exist in an endogenous state of dynamical criticality, characterized by a greater than random probability of both prolonged periods of phase-locking and occurrence of large rapid changes in the state of global synchronization, analogous to the neuronal “avalanches” previously described in cellular systems. Moreover, evidence for critical dynamics was identified consistently in neurophysiological systems operating at frequency intervals ranging from 0.05–0.11 to 62.5–125 Hz, confirming that criticality is a property of human brain functional network organization at all frequency intervals in the brain's physiological bandwidth.

## Author Summary

Systems in a critical state are poised on the cusp of a transition between ordered and random behavior. At this point, they demonstrate complex patterning of fluctuations at all scales of space and time. Criticality is an attractive model for brain dynamics because it optimizes information transfer, storage capacity, and sensitivity to external stimuli in computational models. However, to date there has been little direct experimental evidence for critical dynamics of human brain networks. Here, we considered two measures of functional coupling or phase synchronization between components of a dynamic system: the phase lock interval or duration of synchronization between a specific pair of time series or processes in the system and the lability of global synchronization among all pairs of processes. We confirmed that both synchronization metrics demonstrated scale invariant behaviors in two computational models of critical dynamics as well as in human brain functional systems oscillating at low frequencies (<0.5 Hz, measured using functional MRI) and at higher frequencies (1–125 Hz, measured using magnetoencephalography). We conclude that human brain functional networks demonstrate critical dynamics in all frequency intervals, a phenomenon we have described as broadband criticality.

## Introduction

Critical dynamics are recognized as typical of many different physical systems including piles of rice or sand, earthquakes and mountain avalanches. Dynamic systems in a critical state will generally demonstrate scale-invariant organization in space and/or time, meaning that there will be similar fluctuations occurring at all time and length scales in the system. In other words, there is no characteristic scale to critical dynamics, which will be optimally described by scale-invariant or fractal metrics. Thus, power law or fractal scaling has been widely accepted as a typical empirical signature of non-equilibrium systems in a self-organized critical state [1], although the existence of power law scaling does not by itself prove that the system is self-organized critical (SOC). For example, turbulence is a conceptually distinct class of dynamics, which is also characterized by self-similar or scale-invariant energy cascades, that can be empirically disambiguated from criticality [2],[3].

**Figure 1:**
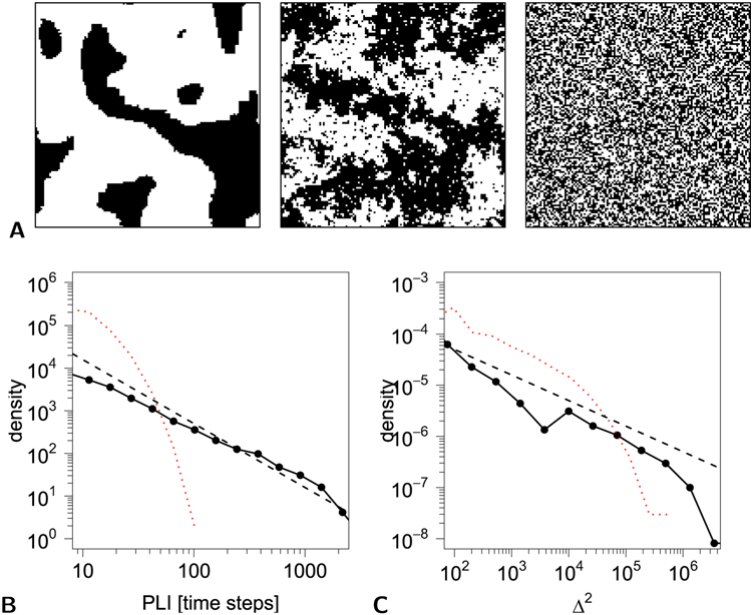
Ising model simulations of a dynamic system at critical and non-critical temperatures. (A) Binary 128×128 lattices showing the configuration of spins after 2,000 timesteps at low temperature, 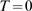 (left); critical temperature, 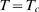 (middle); and high temperature, 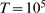 (right). At hot temperature the spins are randomly configured, at low temperature they are close to an entirely ordered state, and at critical temperature they have a fractal configuration. (B) Probability distribution of phase lock interval (PLI) between pairs of processes at critical (black line) and at hot temperature (red line) plotted on a log-log scale. The black dashed line represents a power law with slope 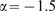. (C) Probability distribution of lability of global synchronization (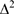) at critical temperature (black line) and at hot temperature (red dotted line); the black dashed line represents a power law with slope 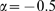. For the cold Ising model the equilibrium state of the system is a monolithic lattice with either all spins up or down, resulting in an entirely static system for which the PLI distribution is a Dirac Delta peak at the duration of the time series. The key point is that the probability distributions of both duration of pairwise synchronization, indexed by the phase lock interval, and lability of global synchronization, show power law behaviour for the 2D Ising model specifically at critical temperature.

**Figure 2:**
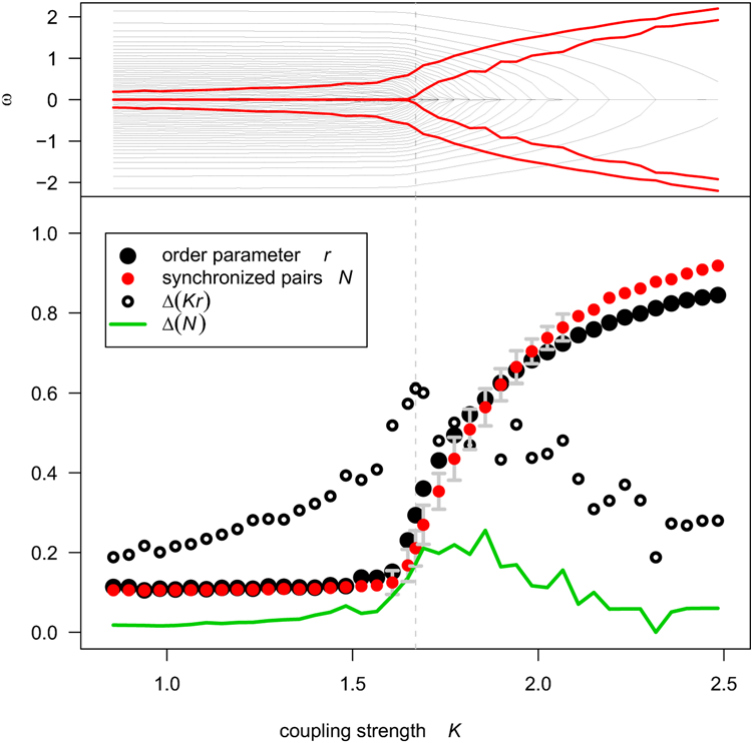
Kuramoto model simulations of dynamic system states as functions of coupling strength between oscillators. When the coupling strength has critical value 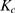, the system is metastable and demonstrates the greatest fluctuations in the mean field and in the number of synchronized pairs. Top panel: change of effective frequencies of oscillators (black lines) with coupling strength 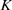 (equivalent to natural frequencies for low 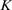). Vertically symmetric red bands indicate range over time of effective mean-field coupling strength 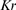. Natural frequencies lower than 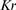 synchronize with the mean frequency 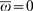, leading to variations in fraction of synchronized pairs subject to fluctuating mean-field strength 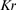. Bottom panel: dependence on coupling strength 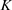 of time averaged order parameter 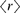 (black circles), and of fraction of synchronized oscillators 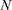 (red circles) with standard deviation indicated by error bars in gray. The open black symbols and green curve show fluctuation amplitudes of 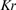 and 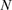, respectively.

The existence of power laws for the spatial and temporal statistics of critical systems is compatible with the related observations that the dynamics of individual units or components of such systems will show long-range correlations in space and time, and change in state of a single unit can rapidly trigger macroscopic reconfiguration of the system. Many of these phenomena can be studied using computational models of dynamic systems such as the Ising model of magnetization (see Figure 1) and the Kuramoto model of phase coupled oscillators (see Figure 2). In both these models, the dynamics can be controlled by continuous manipulation of a single parameter. For the Ising model, this control parameter is the temperature; whereas for the Kuramoto model it is the strength of coupling between oscillators. In both cases, as the control parameter is gradually increased (or decreased), the dynamics of the systems will pass through a phase transition, from an ordered to a random state (or vice versa), at which point the emergence of power law scaling and other fractal phenomena will be observed at the so-called critical value of the control parameter. Self-organized critical systems differ from these computational models in the sense that they are not driven to the cusp of a phase transition by external manipulation of an control parameter but instead spontaneously evolve to exist dynamically at that point.

Self-organized criticality is an intuitively attractive model for functionally relevant brain dynamics [4]–[7]. Many cognitive and behavioral states, including perception, memory and action, have been described as the emergent properties of coherent or phase-locked oscillation in transient neuronal ensembles [8]–[11]. Critical dynamics of such neurophysiological systems would be expected to optimize their capacity for information transfer and storage, and would be compatible with their rapid reconfiguration in response to changing environmental contingencies, conferring an adaptive ability to switch quickly between behavioral states [12].

In support of the criticality model for brain dynamics, there is already considerable evidence for fractal or power law scaling of anatomically localized neurophysiological *processes* - including spike frequency, synaptic transmitter release, endogenous EEG and fMRI oscillations [13]–[15] - measured on a wide range of spatial and time scales. However, there have been fewer direct demonstrations of critical dynamics of anatomically distributed neurophysiological *systems*. Beggs, Plenz and colleagues [16]–[18] have provided empirical evidence of criticality for neuronal network dynamics, represented by a power law probability distribution for the number of electrodes simultaneously recording spike activity in multielectrode array recordings of cortical slices, consistent with the fairly frequent occurrence of neuronal “avalanches”. At the much larger spatial scale of human magnetoencephalography (MEG), the topology of small-world human brain functional networks was found to be self-similar over a range of frequency scales, and the network's topology at each scale was consistent with dynamics close to the critical point of transition from macroscopically chaotic to ordered states [12]. Here we provide more direct evidence for critical dynamics of human brain functional networks measured using both functional magnetic resonance imaging (fMRI) and MEG.

We focused on two measures of the phase synchronization between component processes of a dynamic system (which are defined more formally later): the phase lock interval (PLI) and the lability of global synchronization. The phase lock interval is simply the length of time that a pair of bandpass filtered neurophysiological signals, simultaneously recorded from two different MEG sensors or two different brain regions in fMRI, are in phase synchronization with each other. Thus it is a measure of functional coupling between an arbitrary pair of signals in the system. The lability of global synchronization is a measure of how extensively the total number of phase locked pairs of signals in the whole system can change over time. A globally labile system will experience occasional massive coordinated changes in coupling between many of its component elements. In this sense, global lability is informally analogous to the measure of neuronal “avalanches” introduced by Beggs & Plenz (2003) to describe simultaneous spiking of large numbers of cells in a multielectrode array measurement of spontaneous neuronal activity.

In order to calibrate the behavior of these two synchronization metrics in relation to unquestionably critical dynamics, we first applied them to analysis of the Ising and Kuramoto models as their control parameters were manipulated systematically. These preliminary analyses of two mechanistically distinct computational models demonstrated that the probability distributions of both synchronization metrics followed a power law specifically when the models were in a critical state. This suggested that power law scaling of network synchronization was indicative of critical dynamics regardless of differences in the mechanistic interactions between components in the two models. On this basis, we proceeded to investigate the behavior of these synchronization metrics in neurophysiological data recorded from healthy human volunteers using functional MRI and MEG.

## Methods and Materials

### Synchronization Metrics and Scaling in Critical Models

#### Scale-dependent phase synchronization

To calculate a locally time-averaged estimate of the phase difference between two time series, 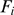 and 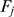, we used their wavelet coefficients derived using Hilbert transform pairs [19]. For a comprehensive review of methods to characterize bivariate relationships between time series, especially the relevant prior work by Lachaux et al. [20],[21], see Pereda et al. [22].

In this work the instantaneous complex phase vector is defined as:
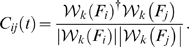
(1)Here 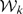 denotes the 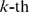 scale of a Hilbert wavelet transform, and ^†^ denotes the complex conjugate. In order to get a less noisy estimate of the phase it is beneficial to average over a brief period of time using a sliding window technique. Mathematically this is equivalent to:
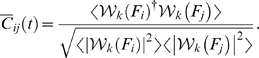
(2)averaging 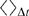 over the interval 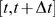 with 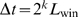. The window size 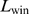 expressed in units of wavelet-scale determines the number of cycles over which the average is taken and was chosen as 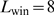 here. This means that 8 oscillations of the highest frequency sampled by a particular wavelet scale are used for the average and, since the wavelet width is one octave, 4 oscillations of the lowest frequency.

The argument of 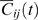, henceforth denoted 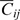 for simplicity, is then the local mean phase difference between the two signals 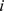 and 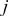 in the frequency interval defined by the 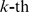 wavelet scale, i.e.,
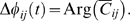
(3)Moreover, the modulus squared of the complex time average 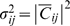 provides a direct measure of the significance of this phase difference estimate. To see this, we note that 
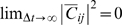
 for independent phases and 1 when there is complete phase locking. In fact 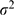 is formally equivalent to the definition of classical coherence, with Fourier coefficients replaced by wavelet coefficients.

We also note that 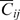 is very similar to the standard phase synchronization index gamma described by Pikovsky et al. [23]. Specifically, gamma is equivalent to 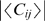, the modulus of the time-windowed moving average of our metric 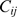, as defined in Equation 1. However, we decided to perform the averaging in a slightly different way, as shown in Equation 2, such that phase vectors with larger amplitude were given greater weight in the average. This refinement of the standard gamma metric improves its robustness against phase interference inherent in the rather noisy experimental data.

Intervals of phase-locking, or phase synchronization, can be defined as periods when 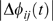 is smaller than some arbitrary value. Here we will define the two processes as phase-locked or synchronized when 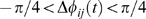, and the duration of phase locking, or phase locked interval, is the length of time for which this condition holds true. The threshold value of 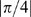 was chosen because it represents the mid-point between exact synchronization 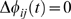 and complete independence 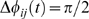 or 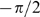. (Note that phase differences 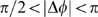 denote various degrees of *anti*-correlation rather than independence.) Additionally we require 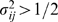, limiting our analysis to phase difference estimates above this level of significance.

#### Global lability of synchronization

Given estimates of the phase difference between each pair of signals in the system, it is then possible to count the number of pairs of signals that are phase-locked at any point in time:
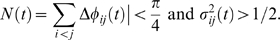
(4)This provides a global measure of the extent of synchronization in the system. We can also calculate the difference in the number of synchronized pairs at two points in time:
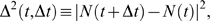
(5)choosing a value of 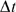 larger than the window size 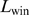 used to calculate the phase difference. This provides a measure of the lability of global synchronization of the system. Large values of 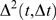 indicate extensive change in global synchronization.

### Computational Models

#### The Ising model

The Ising model [24] was originally defined as a 1D model of ferromagnetism but has since been extended in generality to two and higher dimensions [25]. Recently it has also become widely used as a paradigmatic example of critical dynamics in a relatively simple system [26]. We defined a 2D Ising model operationally as follows. In a square 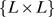 lattice, each one of 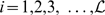 sites was associated with a variable or “spin”, 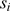, with one of two possible values, +1 (an up spin) or −1 (a down spin). Thus any particular configuration of the lattice was completely specified by the set of variables 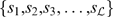. The energy of the system is given by
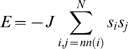
(6)where 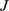 is the coupling constant and the sum of 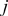 runs over the nearest neighbors 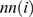 of a given site 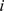. At a given point in time, a spin can flip from one possible state to another if it is energetically favorable but also if it is not, with the probability 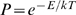, where 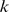 is Boltzmann's constant and 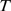 is the temperature (analogous to an actual physical system). The simulation was implemented with the Metropolis Monte Carlo algorithm solving for a given temperature 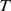. In the case of the 2D Ising model the critical temperature 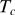 is defined [27] by the equation
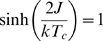
(7)or equivalently 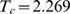 if we choose units such that 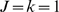 without loss of generality.

We instantiated this model in a {96×96} lattice at three different temperatures: 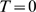 (cold), 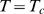 (critical) and 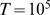 (hot). Our objective was to estimate instantaneous phase differences between each pair of signals (Equation 3), and the lability of global synchronization (Equation 5), in these simulations to provide a point of reference for comparable analysis of neurophysiological data. To produce time series that were continuously variable in the range [−64,64], rather than binary, the magnetization was averaged over local neighborhoods or square {8×8} sub-lattices at each time point, resulting in 144 continuous time series. Each simulation was initiated with the spins in a random configuration and iterated for 12,192 time steps. At low temperatures it will take the system some time to reach its equilibrium state and we therefore restricted our analysis to the final 8,192 timepoints of each simulation.

In the simulated data from the 2D Ising model at critical temperature, we found that the probability distributions for both the phase-lock interval (PLI) and global lability (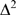) demonstrated power law scaling specifically when the system was at critical temperature; see Figure 1.

#### The Kuramoto model

The 2D Ising model is one of the simplest computational models available for studying critical dynamics, which is its main advantage. However, the physical mechanism on which it is based, magnetic coupling of neighbouring spins in a ferromagnetic material, and the extreme simplicity of its components, binary spins, may seem to be implausibly related to the components and mechanisms of brain networks. We therefore also implemented the Kuramoto model as an alternative, independent model of critical dynamics. This seemed a natural choice since our measures of network dynamics are based on phase synchronization, and the Kuramoto model describes the phase evolution of its elements explicitly. It is also a parsimoniously simple system, yet able to produce a number of surprisingly complex phenomena. In particular, it will undergo a second order phase transition when the coupling parameter is in the vicinity of its critical value 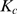, analogous to the critical temperature in the Ising model. The Kuramoto model has been widely used to study synchronization phenomena in complex dynamical systems [28] arising in many different contexts ranging from physics to biology. For example, it has been applied to the neurophysiological problem of stimulus integration in sensory processing in neural networks [8],[29] and also to the study of intermittent dynamics in EEG data [30].

In the Kuramoto model, the system is comprised of 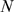 limit-cycle oscillators each of which has its own natural frequency 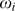, and is also coupled to all other oscillators in the system through a periodic function of the pairwise phase difference 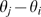, such that the differential equation for the evolution of the phase of a given oscillator 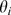 is:
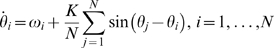
(8)where 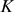 denotes coupling strength. The distribution of natural frequencies 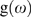 can be chosen freely but is usually limited to being unimodal and symmetric about its mean 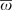. Moreover, without loss of generality, we can transform the coordinate system into a comoving frame, rotating at 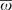, such that the effective mean frequency becomes 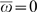.

For our simulations, we selected a set of 44 normally distributed frequencies with zero mean and unit variance 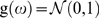. As demonstrated analytically by Kuramoto [31], the critical coupling exponent 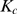 does not depend on the exact shape of 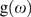, but is solely a function of the probability density at the central frequency 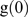:
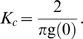
(9)With 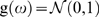 this would formally give 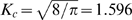, but since we used a discrete frequency distribution rather than a continuous one, we calculate the probability density independently using a smoothing kernel approach. This gives a slightly different result, depending on the exact set of natural frequencies 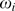.

It is convenient to introduce a global order parameter 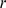 as the modulus of the complex mean over all phase vectors
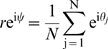
(10)where 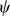 is the mean phase. With this definition Equation 8 can be rewritten in terms of coupling to the *mean field*:
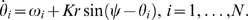
(11)In this form, the equation for the phase evolution in the model becomes more intuitive. In particular, under the assumption that the mean field reaches a stationary equilibrium in the limit 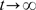, then 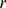 and 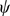 become invariant and the differential equations decouple completely. This is how Kuramoto initially solved the model analytically. However, we are not interested in the model when it is in a quasi-stationary state but rather when it is in an unstable or metastable state, which is the case when the coupling strength is at the critical value 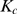.

This can be seen in Figure 2, which illustrates the rapid change of the system states when the coupling strength exceeds 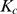. The point of critical coupling strength is marked by the greatest fluctuation in the number of synchronized pairs, and the greatest range of 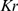, the strength of effective coupling to the mean field (see Equation 11). Consequently the oscillators whose effective frequencies lie within this range experience intermittent periods of strong and weak driving by the mean field, pushing them in and out of synchronization, resulting in a chaotic system. The evolution of each individual oscillator is thus dynamically equivalent to a circle-map oscillator, a prototypical chaotic system [32].

To generate time series from the Kuramoto model in critical and non-critical states, we simulated the phase evolution of a set of 44 coupled oscillators (with natural frequencies specified as described above) and solved the set of 44 coupled evolution equations (Equation 8) numerically using ODE solvers which distinguish automatically between stiff and non-stiff problems [33],[34]. Each simulation ran for 10^5^ time steps, which were selected to be sufficiently small to sample the highest frequencies in the model accurately with at least 8 values per cycle. Two sets of time series were produced: one with the coupling parameter set at its critical value 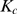 and one with 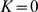, i.e. free running oscillators.

**Figure 3:**
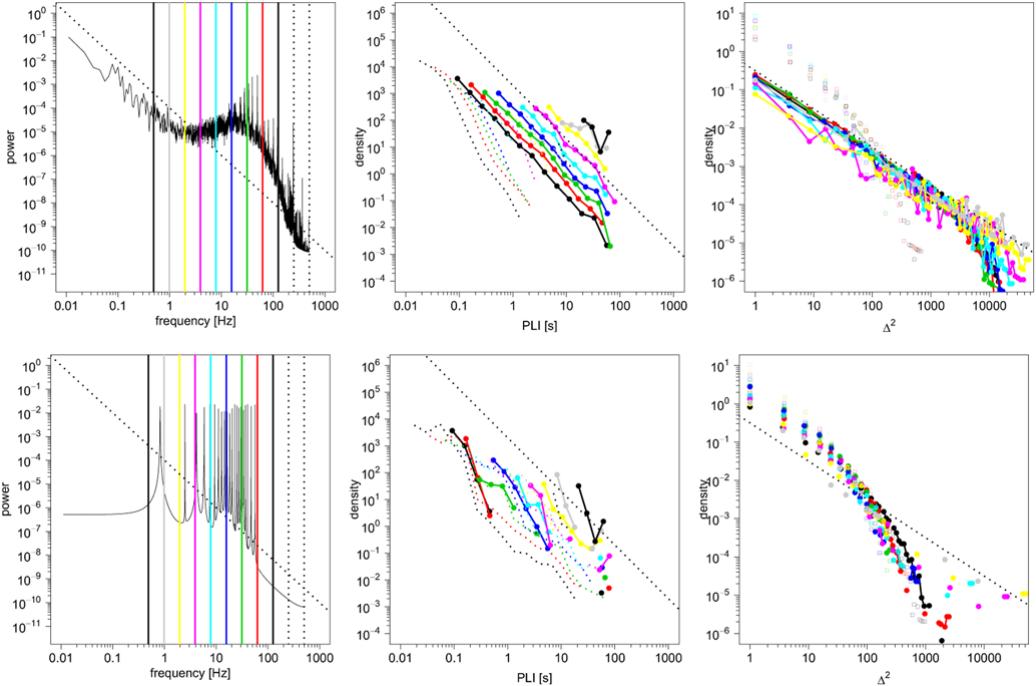
Simulated Kuramoto model data. Top row: results from system at critical coupling strength 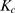, bottom row: no coupling, i.e. free running oscillators. In all panels simulation data is denoted by solid lines (filled symbols) and the corresponding surrogate data by dotted lines. The colors encode wavelet scales 3–11. Left column: Power spectrum of simulated Kuramoto model time series plotted on logarithmic axes. In the critical state the spectrum shows clustering of the effective frequencies forming a common broad peak and follows a power law with exponent −2 on the low-frequency end. The spectrum of the uncoupled model is a simple superposition of the natural oscillator frequencies. The colored vertical lines represent the frequency intervals corresponding to wavelet scales 3–11 (scales 1 and 2 indicated by dotted lines not used). Middle column: Probability distributions for phase-lock interval PLI. Only the critical system produces a power law, clearly distinct from the surrogate data showing an exponential fall-off. The black dashed line represents a power law with 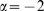. Right column: Probability distribution for lability of global synchronization 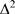 is plotted on logarithmic axes for each wavelet scale. Again a power law is only seen in the critical model, whereas surrogate data and uncoupled model produce exponential distributions. The straight dashed line represents a power law with 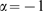.

In the simulated data from the Kuramoto model, we found that the probability distributions for both the phase-lock interval (PLI) and global lability (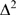) demonstrated power law scaling specifically when the system was at critical coupling strength; see Figure 3. The goodness-of-fit for a power law probability distribution of PLI was compared, using Akaike's information criterion (AIC), to the goodness-of-fit for exponential and log normal distributions. We used a variant of the AIC including a second order correction for small sample sizes, defined as
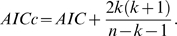
(12)where 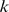 is the number of parameters in the model, and 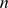 is the number of observations in the data [35].

The AIC is designed to identify the fit which best explains the data with a minimum of free parameters. As shown in Table 1, the power law distribution quite consistently provided the best fit over all wavelet scales.

**Table 1:** Power law scaling of phase lock interval (PLI) probability distributions. Akaike goodness-of-fit criterion for various fitting functions applied to the PLI distributions of the Kuramoto model and the fMRI and MEG data, respectively.

Kuramoto Model	Power-Law	Exponential	Log-Normal
125−62.5 Hz	−11.4	48.3	9.2
62.5−31 Hz	−20.7	40.2	3.0
31−15.5 Hz	12.1	36.1	6.6
15.5−8 Hz	−8.5	32.9	5.9
8−4 Hz	−0.9	23.6	6.8
4−2 Hz	2.3	19.0	2.1
2−1 Hz	−2.0	11.1	0.4
1−0.5 Hz	15.7	13.4	15.0
0.5−0 Hz	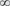	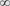	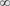

MEG Data	Power-Law	Exponential	Log-Normal
125−62.5 Hz	0.8	28.0	5.5
62.5−31 Hz	−6.8	31.3	5.4
31−15.5 Hz	−6.5	35.5	5.4
15.5−8 Hz	−2.1	28.8	3.6
8−4 Hz	−0.0	34.2	12.9
4−2 Hz	−5.3	31.6	9.7
2−1 Hz	−4.7	24.1	6.6
1−0.5 Hz	6.7	19.9	12.2

fMRI Data	Power-Law	Exponential	Log-Normal
0.45−0.22 Hz	−15.7	50.7	13.7
Intra-modular	−7.2	60.7	17.4
0.22−0.11 Hz	−0.5	18.0	−9.5
Intra-modular	−18.1	18.6	−10.8
0.11−0.05 Hz	−7.7	9.7	0.4
Intra-modular	−10.3	5.3	−3.9

Smaller values indicate a better fit, but comparisons are only meaningful across rows.

### Acquisition and Preprocessing of Experimental Data

All experimental studies on human subjects were conducted according to the principles of the Declaration of Helsinki and the standards of Good Clinical Practice. All participants provided informed consent in writing. The study protocols were ethically approved by the Addenbrooke's NHS Trust Research Ethics Committee and the Cambridgeshire 2 Research Ethics Committee, Cambridge UK.

#### Functional MRI

A group of 17 healthy volunteers (aged 18–33 years, mean = 24.3 years) was scanned, lying quietly at rest with eyes closed for 9 min, 37.5 s [36]. Gradient-echo echoplanar imaging (EPI) data depicting BOLD contrast were acquired using a Medspec S300 scanner (Bruker Medical, http://bruker-medical.de) operating at 3.0 T in the Wolfson Brain Imaging Centre (Cambridge, UK). 525 volumes were acquired with the following parameters: number of slices, 21 (interleaved); slice thickness, 4 mm; interslice gap, 1 mm; matrix size, 64×64; flip angle, 90°; repetition time (TR), 1100 ms; echo time, 27.5 ms; in-plane resolution, 3.125 mm. The first seven volumes were discarded to allow for T1 saturation effects, leaving 518 volumes available for analysis.

Each dataset was corrected initially for geometric displacements because of head movement and co-registered with the Montreal Neurological Institute gradient-echo echoplanar imaging (EPI) template image, using an affine transform implemented in SPM2 software (http://www.fil.ion.ucl.ac.uk). Two datasets that had been affected by head movement in excess of 3 mm translation, or 0.3° rotation, in x, y, or z dimensions, were discarded. The remaining data were not spatially smoothed before regional parcellation using the anatomically labeled template image validated previously by Tzourio-Mazoyer et al. [37]. This parcellation divides each cerebral hemisphere into 45 anatomical regions of interest. Regional mean time series were estimated for each individual by averaging the fMRI time series over all voxels in each of 90 regions. Each regional mean time series was further corrected for the effects of head movement by regression on the time series of translations and rotations of the head estimated in the course of initial movement correction by image realignment [38]. The residuals of these regressions constituted the set of regional mean time series used for wavelet-based synchronization analysis. Each region was additionally assigned to a functionally-related cluster or module based on a prior hierarchical cluster analysis of resting-state fMRI data on an independent sample [39].

#### Magnetoencephalography

Data were acquired from two healthy subjects, sitting quietly with the instruction to keep their eyes shut for 3.5 minutes. MEG data were continuously sampled at a frequency of 1000 Hz by 204 planar gradiometers and 102 magnetometers in an Elekta Neuromag MEG scanner at the MRC Cognition and Brain Sciences Unit (Cambridge, UK). Head position, horizontal and vertical electrooculogram were continuously monitored throughout recording. The data set was corrected for the presence of internal and external noise sources and for disturbances as a result of head movements using signal space separation [40] with the spatiotemporal extension [41] via MaxFilter (Elekta Neuromag, Finland). Bad channels were removed from the data set prior to applying signal space separation and interpolated from neighbouring sensors afterwards.

## Results

### Neurophysiological Systems

#### Phase-locking and lability of global synchronization in functional MRI

**Figure 4:**
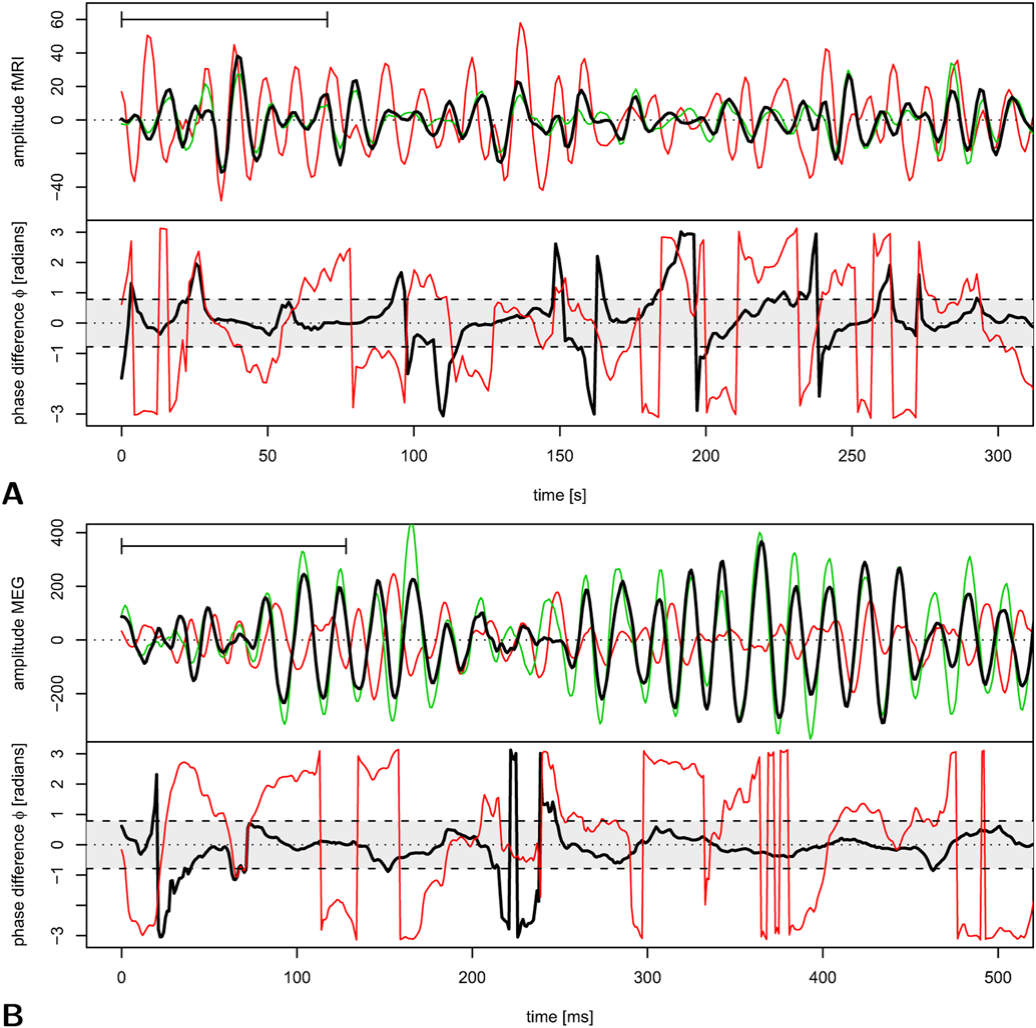
Illustration of phase synchronization between pairs of neurophysiological processes in low and high frequency intervals. (A) Top panel: Amplitude of functional MRI signals in the frequency interval 0.05–0.1 Hz (corresponding to wavelet scale 3) is shown for three brain regions in a single subject: left precentral gyrus (black), right precentral gyrus (red), and left olfactory cortex (green). Bottom panel: Phase difference between two pairs of fMRI processes is shown for right and left precentral gyrus (black), and left precentral gyrus and olfactory cortex (red). The shaded area represents phase differences less than 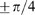; while phase difference is in this regime the pair of processes is said to be phase-locked. (B) Top panel: Amplitude of MEG signals in the frequency interval 31–63 Hz (approximately equivalent to the classical 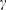 band) is shown for three sensors in a single subject: two left temporal sources (black and green), and a left frontal source (red). Bottom panel: Phase difference between two pairs of MEG processes is shown for 2 left temporal sensors (black) and for left frontal and temporal sensors (red). The horizontal bars in the top-left corner of each panel denote the temporal extent of 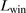, respectively, corresponding to about 8 wavelet cycles. Only a short section of the actual time-series is shown.

Periods of phase-locking between pairs of regional fMRI time series were estimated using the Hilbert transform of their wavelet coefficients at scales 1, 2, and 3 of the discrete wavelet transform (corresponding to an overall frequency range of 0.05–0.45 Hz). As illustrated in Figure 4, showing a short segment of signals from the scale 3 frequency interval (0.05–0.11 Hz), this procedure results in a continuously variable estimate of the locally time-averaged phase of each time series 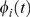, and the phase difference between each pair of time series 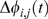. Periods of phase synchronization or phase-locking were defined as time intervals when the phase difference was arbitrarily close to zero, i.e., 
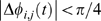
.

**Figure 5:**
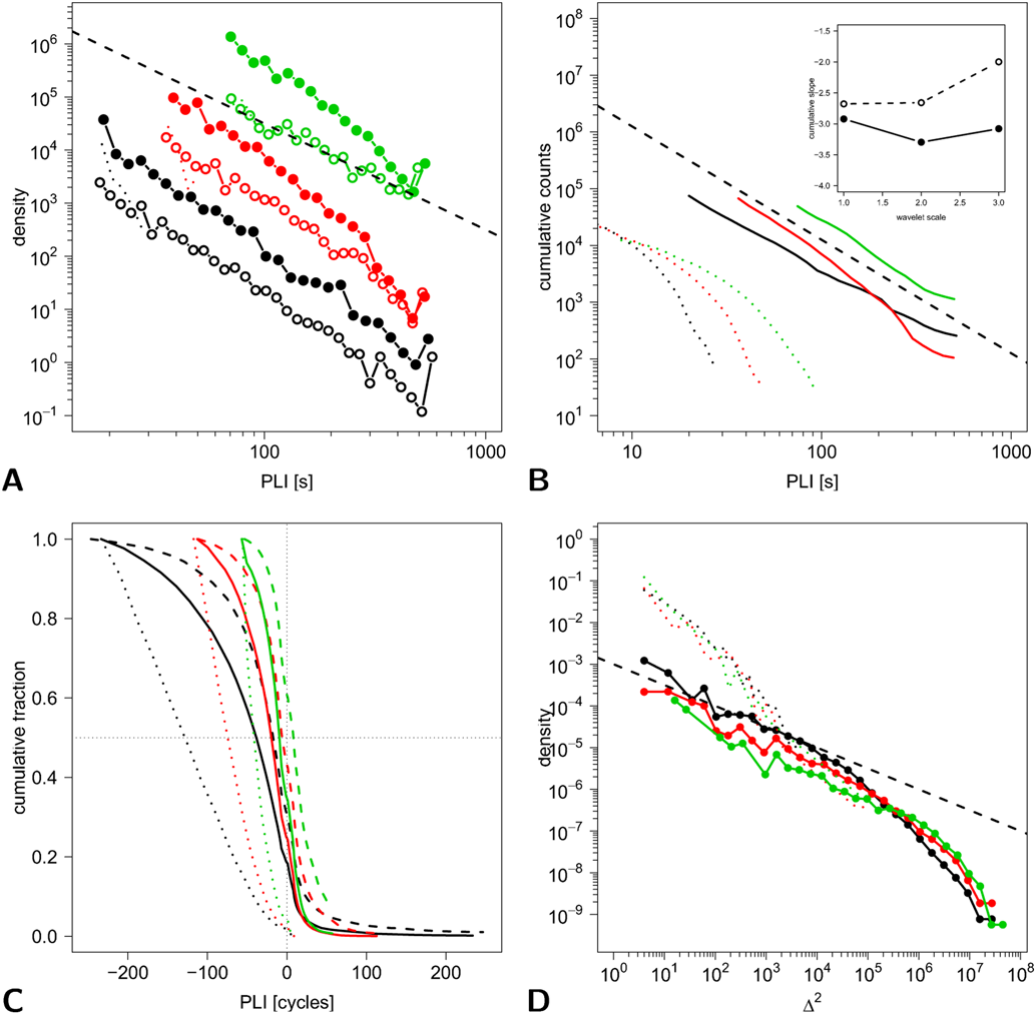
Phase-locking and global synchronization in a low frequency network measured using functional MRI. Colors denote wavelet scales: black  =  scale 1 (0.45−0.22 Hz); red  =  scale 2 (0.22−0.11 Hz); green  =  scale 3 (0.11−0.05 Hz). (A) Probability distributions of phase-lock interval (PLI, s) are plotted on logarithmic axes for all pairs of processes (filled symbols) and for all (intra-modular) pairs of processes within the same functional module (open symbols). The corresponding distributions for phase-scrambled surrogate data are shown by the dotted lines, and the straight dashed line indicates a power-law with 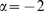. (B) Cumulative probability distributions of phase-lock intervals are shown on logarithmic axes for all pairs of processes (solid lines) and surrogate data (dotted lines). Inset shows the power law scaling exponent 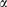 as a function of wavelet scale (larger scales represent lower frequencies). (C) Weighted cumulative probability distribution of phase-lock intervals are shown on linear axes for all pairs of processes (solid lines), intra-modular pairs of processes (dashed lines) and surrogate data (thin dotted lines). The negative range on the x-axis stands for intervals without phase-lock. (D) Probability distributions for lability of global synchronization (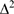) are shown on logarithmic axes for fMRI data (filled symbols and solid lines) and surrogate data (dotted lines). The dashed straight line indicates a power law with 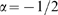 to guide the eye.

We can see immediately that there is variability in the extent of phase-locking between different pairs. The bilaterally homologous pair of regions (right and left precentral gyrus) show more prolonged periods of phase-locking than the anatomically unrelated pair of regions (left precentral gyrus and olfactory cortex). Moreover, all pairs of signals show periods of phase-locking interspersed with periods of phase independence. The intermittency of phase-locking was quantified by plotting the empirical probability distribution of the phase-lock interval (PLI) over all pairs of signals in the image. As shown in Figure 5, this distribution followed a power law, i.e., 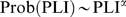, with the power law exponent 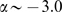 (its precise value depending on scale; see inset of Figure 5B). The goodness-of-fit for a power law probability distribution of PLI was compared, using AIC, to the goodness-of-fit for exponential and log normal distributions. As shown in Table 1, the power law distribution quite consistently provided the best fit over all wavelet scales.

Considering separately those pairs of signals within the same functional module of the network, we found that pairwise intra-modular synchronization also followed a power law distribution for phase-lock interval but the scaling exponent was considerably smaller for the intra-modular distribution (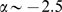), indicating that the probability of long periods of phase synchronization was greater for intra-modular pairs. In contrast, the probability distribution of phase-lock interval for pairs of surrogate signals, created by randomly permuting the phases of the original fMRI signals in the Fourier domain, was better described by an exponential than by a power law. A complementary perspective is provided by plotting the cumulative probability distributions for the fMRI and surrogate data, weighted by the time duration of phase locking, which provides a clearer indication of the periods of time spent in phase-synchronized or phase-incoherent states; see Figure 5C.

**Figure 6:**
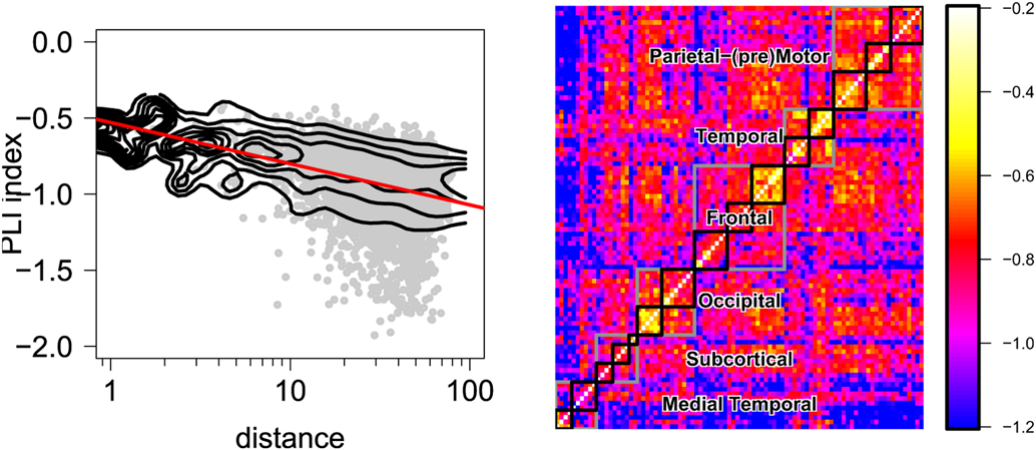
Effects of spatial proximity and modularity on scaling of phase locking between fMRI time series. Left: Dependence of phase-lock index on log of physical distance between a pair of brain regions (dots). The number density is indicated by contours, where the change in abundance with distance has been normalized out. The red line has a slope of 0.25 and serves to guide the eye. Right: Matrix representing the relative prevalence of long-term phase lock versus short-term phase lock intervals for all pairs of brain regions in resting-state fMRI data. The color of each element indicates the value of 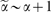 for a specific pair of processes (see text for exact definition of 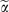). Intra-modular pairs of regions are located close to the diagonal and are segregated and identified by black rectangles (grey rectangles denote coarser anatomical separation of brain regions as labeled; see text). All graphs shown are for wavelet scale 1 in the fMRI data.

For a finer-grained representation of the variability of phase synchronization between different pairs of 90 regional fMRI time series, we calculated the relative prevalence of long-term lock versus short-term lock duration as 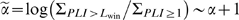 for all possible pairs and collated the results in a {90×90} matrix; see Figure 6. (For individual pairs we have only very small number statistics and 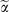 is more robust than a direct fit of 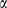). This analysis confirms that intra-modular connections between regions are typically associated with smaller absolute values of 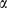, reflecting greater probability of long intervals of phase-locking between regions belonging to the same functional module (module definition according to [39]; in particular see their Figure 3). In fact a number of connections inside these modules were locked for the whole length of the analyzed timeseries in a majority of subjects (yellow and white matrix elements in Figure 6), causing the excess for the maximal PLI in the distribution shown in Figure 5A.

Even though this paper does not primarily deal with topological analysis, it should be noted that functional network properties calculated by thresholding this matrix of pairwise phase-locking were consistent with results previously reported by Achard et al. [42], who found an exponentially truncated power law for the degree distribution in an independent sample of fMRI data. The network hubs in these data were mostly regions of association cortex with connections predominantly to other nodes in the same functional modules. In particular the dorsal superior frontal gyrus bilaterally was distinguished by high degree and numerous inter-modular connections.

The probability distribution for 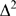, the lability of global synchronization, is also represented on logarithmic axes in Figure 5D. This distribution approximates closely to a power law, whereas the equivalent distribution calculated for surrogate data does not. This result indicates that large changes in the number of simultaneously phase-locked processes are more likely to occur in functional MRI data than would be expected under the null hypothesis.

#### Phase-locking and lability of global synchronization in MEG

For a representative MEG time series, the signal amplitude in wavelet scale 4 (corresponding to a frequency interval of 31–62.5 Hz) is shown for three representative sensors in Figure 4. Also shown are the time-localized estimates of phase difference between two pairs of these sensors.

**Figure 7:**
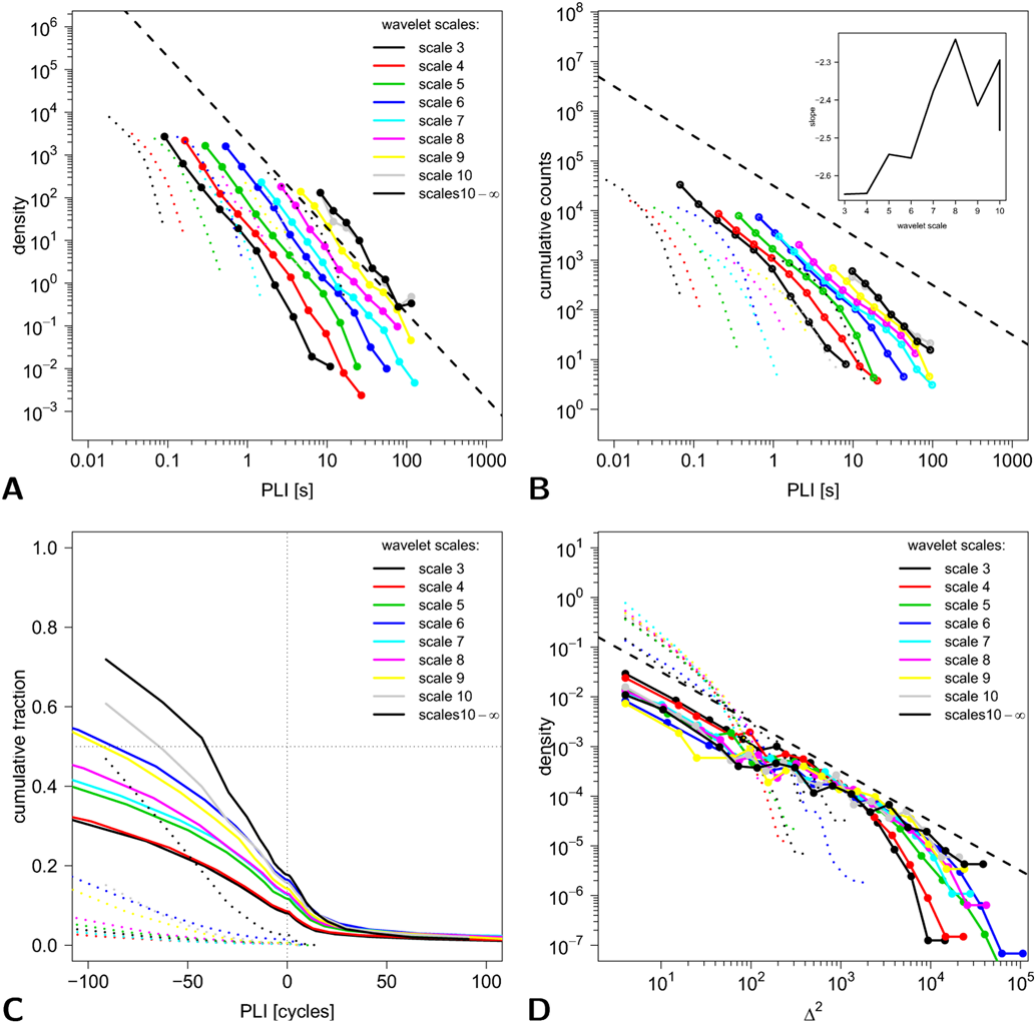
Probability distributions for phase-lock interval and lability of global synchronization in MEG data. In all panels MEG data is denoted by solid lines (filled symbols) and the corresponding surrogate data by dotted lines. The colors encode wavelet scales as follows: black  =  scale 3 (125−62.5 Hz); red  =  4 (62.5−31 Hz); green  =  5 (31−15.5 Hz); blue  =  6 (15.5−8 Hz); light blue  =  7 (8−4 Hz); pink  =  8 (4−2 Hz); yellow  =  9 (2−1 Hz); gray  =  10 (1−0.5 Hz); black  =  11 (0.5−0 Hz). (A) Probability distribution of phase-lock intervals is plotted on logarithmic axes for each wavelet scale. The black dashed line represents a power law with 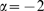. (B) Cumulative probability distribution of phase-lock intervals is plotted on logarithmic axes for each wavelet scale. The exponents of the power law distributions tend to increase as a function of increasing scale (inset). (C) Weighted cumulative distribution of phase-lock intervals on linear axes for each wavelet scale indicating the fraction of time spent locked for a time longer than the PLI indicated. (D) Probability distribution for lability of global synchronization 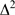 is plotted on logarithmic axes for each wavelet scale. The straight dashed line represents a power law with 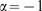.

We generalized this analysis to estimation of phase differences, and phase-lock intervals, for pairs of MEG sensors in each wavelet scale and were thereby able to estimate the scale-specific probability distribution of phase-lock intervals; see Figure 7A. The distributions of PLI approximated a power law reasonably well at all scales, whereas the surrogate data did not. The power law exponent 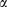, estimated by fitting a straight line to the log transformed density, became increasingly positive as a function of increasing scale (inset of Figure 7B), indicating that the probability of long periods of phase-synchronization tended to be greater in the lower frequency intervals corresponding to larger wavelet scales. This behavior is also represented from a complementary perspective by comparison of the cumulative probability distributions for PLI at each of the wavelet scales (Figure 7C). The goodness-of-fit for a power law probability distribution of PLI was compared, using AIC, to the goodness-of-fit for exponential and log normal distributions. As shown in Table 1, the power law distribution quite consistently provided the best fit over all wavelet scales.

The probability distribution for lability of global synchronization 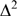 was likewise plotted on logarithmic axes for each wavelet scale; see Figure 7D. The probability of a large change in the number of simultaneously phase-locked processes was generally greater in the MEG data than in comparable surrogate data but power law behavior was evidently limited by the finite size of the system prohibiting very large values of 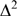.

## Discussion

The aim of this paper was to show by analysis of two mechanistically distinct computational models (the Ising and Kuramoto models) that power law scaling of synchronization metrics is a consistent macroscopic feature of dynamical systems in a critical state; and then to demonstrate that analogous scaling behaviour is a feature also of human brain functional networks measured using functional MRI and MEG. The wavelet-based analysis of fMRI data addressed phase synchronization in low frequency networks oscillating in the interval 0.05–0.45 Hz; whereas the analysis of MEG data addressed higher frequency networks in the interval 1–125 Hz. In both kinds of data, and in all frequency intervals, we found strong evidence for power law scaling of both the phase-lock interval and the lability of global synchronization.

This pattern of results indicates that scaling of synchronization metrics can arise in critical systems regardless of the underlying mechanisms and that broadband criticality is clearly evident in large scale human brain networks derived from substantively different modalities of neuroimaging data. A corollary of these observations is that it is not possible to deduce the mechanism by which criticality emerges in a system simply by measuring these macroscopic behaviors. However, by providing one of the first direct demonstrations that criticality is an emergent property of human brain networks, we hope to motivate future research into the generative mechanisms of criticality in these systems. It would also be important to investigate whether the underlying mechanisms for human brain network criticality are different between fMRI and MEG networks and perhaps also between MEG networks at different frequencies.

### Power Law Scaling

Power law scaling of human neurophysiological processes has been previously described in both functional MRI and MEG or electrophysiological datasets [14],[15],[43]. However, we believe this is the first demonstration of power law scaling of synchronization metrics in human brain networks. It was notable that although power law scaling was demonstrated for all frequency intervals in both fMRI and MEG data, and for all anatomical pairs of regions in the fMRI data, the value of the scaling exponent 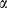 was variable in relation to both the modularity and the frequency interval of the networks. Thus the scaling exponent 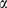 of the PLI distribution was smaller for (intra-modular) pairs of fMRI signals belonging to the same functional module than for (inter-modular) pairs belonging to different functional modules, indicating that prolonged periods of phase locking are generally more likely to occur between functionally related processes. These results are consistent with previous findings that intra-modular pairs of fMRI signals are more strongly correlated than inter-modular pairs [39], probably a direct consequence of their stronger structural connectivity [44], as demonstrated in simulations of hierarchical dynamics on the cortical network of the cat [45]. The dependency of 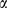 on frequency interval of the networks was most evident by analysis of the MEG data. Here we found that 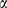 tended to be smaller, indicating a higher probability of long periods of phase locking, in lower frequency networks. This observation is also consistent with prior work demonstrating that wavelet correlations between MEG sensors increase with decreasing frequency in a theoretically predictable way [46]. These considerations suggest that scaling of synchronization metrics, although novel in the context of human neuroimaging, has a profile of variability that makes sense in the context of prior observations on functional brain networks.

However, it is important to bear in mind some methodological caveats when evaluating the empirical demonstrations of power law scaling we have reported. Given that an ideal scale-free system has no well defined limits in space or time, the “lack of infinity” in the data we are studying inevitably has some effect on our results. In particular, the finite length of our time series prevents us from estimating the phase lock interval (PLI) distribution for time intervals longer than about 10 minutes, which will be counted in the last bin of the PLI histogram, corresponding to the duration of the time-series. This will also impact on the estimation of the power law exponent and its impact will be greater for estimation of scaling parameters in lower frequency networks, where we have fewer data points per time series. Therefore we should be cautious about strong interpretations of the absolute value of the estimated power law exponents.

Finite size effects are also clearly visible in the estimated probability distributions of the lability of global synchronization. These are seen not only in the experimental fMRI and MEG data, but also in both computational models, and are a direct consequence of the finite spatial extent of the system in terms of a limited number of pairs. However, it is important to note that the probability distributions of lability are distinct for surrogate data compared to experimental data in all cases. Also, the probability distribution of the number of synchronized pairs 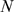 (not shown) for the surrogate data is a narrow Gaussian centered around a small 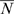, whereas the experimental and simulated data display a much wider non-Gaussian distribution with a comparatively large number of synchronized pairs on average, limited only by the system size.

The importance of finite size effects in interpreting the shape of the empirically estimated probability distributions motivated us to test formally for the goodness-of-fit of a power law distribution (compared to exponential and log normal distributions) for the probability of PLI and lability of global synchronization in the Kuramoto model at critical coupling strength, and both the fMRI and MEG data, at all scales; see Table 1. These results indicate that the power law form is quite consistently the best fitting model for the probability distribution across all datasets and scales.

### Neuroscientific Implications

The added value of this analysis is arguably twofold. First, it indicates that phase synchronization is likely to be an important mechanism of functional network formation at all frequencies and in the endogenous or resting state. Phase synchronization of spatially distributed neurophysiological processes is already accepted as a key mechanism in the transient formation of neuronal ensembles coding the representation of perceived objects or memories [9],[10]. However, most attention has focused on phase synchronization in high frequency intervals, e.g., the gamma frequency band (30–80 Hz), and in response to experimentally controlled stimulation [47]. Our results show that intermittent periods of phase-locking, sometimes for long time intervals, are characteristic of endogenous human brain network dynamics. By analogy to the experimental data demonstrating changes in gamma synchronization in response to conscious perception of external objects, one might speculate that spontaneously occurring periods of phase synchronization might represent changes in subjective mental state, or conscious perception of internal objects. In any case, it is clear from these data that intermittent phase synchronization of neurophysiological systems is a general intrinsic property of the brain and not restricted to certain frequency bands or stimulus conditions.

We would also draw attention to an analogy between the neuronal avalanches previously described in multielectrode array recordings [16]–[18], which represent rapid simultaneous changes in spiking coordinated across a large number of individual neurons, and the scaling behavior of our measure of the lability of global synchronization, which indicates the potential for whole brain systems to demonstrate rapid and extensive changes in global phase locking. This analogy seems consistent with the fundamental principle of scale invariance in understanding critical systems: qualitatively similar network dynamics can be expected at very different (cellular versus whole brain) spatial scales.

The second and main theoretical implication of these results is that they provide direct empirical support for the hypothesis that human brain networks exist dynamically in a critical state. Criticality has been studied most intensively to date in simulated neural networks. These studies indicate that networks at a critical point between order and chaos are optimized for information transmission, and generate a maximum number of metastable global states, conferring a high capacity for information storage [7],[18]. Critical systems rapidly adapt to minimal exogenous perturbation [26], which could have obvious selection advantages for a nervous system. It has also been shown that critical dynamics can emerge by the operation of biologically plausible rewiring rules on initially random networks. For example, a Hebbian rewiring rule, whereby connections are formed between nodes with highly correlated activity (and deleted between nodes with poorly correlated activity), led to the self-organization of critical dynamics in an initially random network [48]. Likewise, when connectivity between neurons was modified by a spike timing dependent plasticity rule, critical dynamics emerged in a functional network with small-world topology [49]. A small-world network is characterized by short average path length between nodes, but large clustering coefficient [50]. This architecture can deliver high efficiency of information processing for low connection costs and is common to many systems such as the internet, the global air transport network and the proteome, as well as the brain. The link between critical dynamics and small-world topology is also implicit in our results, given the strong prior evidence for small-world properties of human brain functional networks derived from fMRI and MEG data [12],[42].

A key, unresolved question concerns the cognitive or mental significance of brain systems criticality. There is very little empirical data directly supporting the important theoretical connection between critical brain dynamics and the adaptivity or versatility of the behavioral repertoire the brain can support. However, it has been reported that changes in the power law scaling exponents of human MEG sensors were highly predictive of success in discriminating low intensity visual stimuli [51], suggesting that critical dynamics can indeed be related to optimal perceptual function. An intriguing study in a substantively different biological system, namely gene expression changes in the macrophage following pathogen challenge, found evidence of critical dynamics in normal intra-cellular signaling and non-critical dynamics in cells that had been behaviorally impaired in their response to pathogens by specific gene knockouts, implying that criticality in this signaling system conferred an adaptivity advantage [52]. A key challenge for future studies will be to define more precisely how the parameters of critical network dynamics, empirically estimated in neuroimaging data, can be related to adaptivity and optimality of human cognitive and behavioral performance.
